# ‘You will never be as good as we are’: a qualitative study of women paramedics’ experiences of sex-based harassment in an Australian ambulance service

**DOI:** 10.29045/14784726.2022.09.7.2.1

**Published:** 2022-09-01

**Authors:** Sally Hanna-Osborne

**Affiliations:** The University of Sydney ORCID iD: https://orcid.org/0000-0002-9715-5185

**Keywords:** gender, paramedic, sex-based harassment

## Abstract

**Objectives:**

Sex-based harassment remains a pernicious and pervasive problem in organisations, as evidenced by the recent #MeToo movement. Little is known about how this issue affects women in the paramedic profession. This study explores the sex-based harassment experiences of women working in a large Australian ambulance service, focusing on harassment from co-workers and managers.

**Methods:**

Long-form, semi-structured interviews were undertaken with women paramedics (n = 30) as part of a larger qualitative study of the careers and work experiences of women paramedics. Interviews were recorded and transcribed verbatim, and thematic data analysis was employed to develop rich descriptions of paramedics’ experiences.

**Results:**

Of the 30 participants, 25 had experienced sex-based harassment from male colleagues. Most commonly this took the form of gender harassment – that is, comments and jokes designed to belittle and demean women on the basis of their gender. Several participants experienced sexualised forms of harassment, including unwelcome sexual attention and propositions. Participants expressed reluctance to report the behaviour through organisational channels because of the perceived futility of doing so and the potential for reprisals and career repercussions. The preferred responses to harassment were informal, and included avoidance, humour, direct appeals and work withdrawal.

**Conclusions:**

Sex-based harassment has a range of damaging consequences for victims and the organisations in which they work. This study is the first to explore how Australian women paramedics experience sex-based harassment in their work. The study has implications for policy and practice to improve gender equality within ambulance services and highlights the need for further research into the extent and nature of the problem across the paramedic profession.

## Introduction

The recent #MeToo movement has brought unprecedented public attention to the problem of workplace sex-based harassment which, despite the decades-long existence of legislation designed to eradicate it, remains pervasive (Australian Human Rights Commission, 2020). Sex-based harassment is defined by Berdahl (2007, p. 641) as ‘behaviour that derogates, demeans, or humiliates an individual based on that individual’s sex’. It is a broader and more useful concept than sexual harassment as, in addition to overtly sexualised behaviours like unwanted sexual attention, it captures hostile conduct of a non-sexualised nature such as sexist put-downs and crude jokes (National Academies of Sciences, Engineering, and Medicine, 2018). Research indicates that this type of harassment, termed *gender harassment*, is by far the most common type of harassment based on sex and can be just as damaging as unwelcome sexual conduct (Langhout et al., 2005; Leskinen et al., 2011).

The significant negative consequences of sex-based harassment are well-established. For targeted individuals, harassment is associated with reduced physical and mental health (Leskinen et al., 2011; Sojo et al., 2016), lower job satisfaction (Willness et al., 2007) and increased job withdrawal (Schneider et al., 1997). For organisations, the costs include lower productivity (Pryor, 1995), increased absenteeism (Schneider et al., 1997) and higher turnover (Sims et al., 2005).

Previous survey-based studies indicate that sex-based harassment is a problem in the paramedic profession, particularly for women. In a recent study of female emergency medical services professionals in the United States, 53% of respondents reported experiencing sexual harassment at some point in their careers (Staats et al., 2021). A study of workplace violence against Australian paramedics found that approximately 38% of female respondents reported experiencing sexual harassment from patients, colleagues or members the public, compared with almost 10% of males (Boyle et al., 2007).

Beyond these statistics, however, little is known about the problem of sex-based harassment in paramedicine and how it impacts those who experience it. Studies of women working in other male-dominated emergency services, including firefighting, policing and the military, suggest that the often ‘hyper-masculine’ cultures within these settings result in heightened hostility towards women, who are perceived as encroaching on men’s occupational territory and violating established notions of gender-appropriate work (Martin & Jurik, 2006; Sasson-Levy, 2011; Tyler et al., 2019). There is also evidence that very few women who experience harassment in masculinised work contexts report the behaviour to a supervisor or through other channels (Firestone & Harris, 2003; Fitzgerald et al., 1995; Pershing, 2003), either because such behaviours may be normalised as an everyday part of workplace interactions (Denissen, 2010; Williams, 1998), or because the woman fears she will be blamed, disbelieved or punished for speaking up (Cortina & Berdahl, 2008; Fitzgerald et al., 1995).

The present study draws on data from a wider qualitative study of the working lives of Australian women paramedics to document and analyse women’s experiences of sex-based harassment from male co-workers and supervisors. It is particularly concerned with understanding the types of harassment that women paramedics were exposed to and the strategies they employed to manage the behaviour and mitigate the harm to their careers and well-being.

## Methods

### Study design and setting

The data for this study come from a doctoral study of the careers and workplace experiences of Australian women paramedics, completed in 2019. In line with the study’s goal of capturing the women’s lived experiences of their work, a qualitative methodology was considered most appropriate. Qualitative research is designed to help us understand the social world from the perspective of the research subject and aims to build rich and nuanced descriptions of complex phenomena by interpreting the meanings people bring to them (Denzin & Lincoln, 2005).

Participants for the study were employed within one public ambulance service in Australia. The service is one of the largest in the world, employing close to 5000 paramedics and providing over 1.2 million ambulance responses annually. At the time of data collection, women represented around 36% of the paramedic workforce, a share which was growing rapidly.

### Data collection

An interview technique was deemed most appropriate as it allowed the women to describe their experiences and interpretations of the social world in their own words. The interview format was semi-structured, allowing the researcher to gather focused information on the specific research questions while affording flexibility to probe interesting and relevant lines of inquiry.

Interviews were conducted in person, by phone or by Skype, depending on the interviewee’s geographic location and preference. Interviews generally lasted from one to two hours and were recorded and subsequently transcribed *verbatim*. A field note was created for each interview which documented the researcher’s impressions and observations and flagged ideas for further consideration and analysis.

### Recruitment and sample

Participants in the study were recruited ‘at arm’s length’, using a sampling strategy which could be described as ‘semi-purposive’ (Huppatz, 2009). An email describing the research was sent by a senior operational manager to all operational employees of the ambulance service, together with a link to an ‘expression of interest’ website created by the researcher. A total of 47 paramedics expressed interest in the study and from these, the researcher purposively selected a sample of 40 to reflect a balanced cross-section of age, length of employment, ranking, specialist qualifications, education, geographic location and full-/part-time status. No male paramedics were included in the sample as the purpose of the research was to explore and understand the specific experiences of women paramedics in the context of a historically all-male occupation.

Interviews were conducted between September 2016 and October 2017. Interviews were ceased after 30 women had been interviewed, based on an assessment that sufficient depth, richness and complexity had been generated by the data in relation to the central research questions (Braun & Clarke, 2021). An overview of the final sample of 30 participants is presented in Table 1.

**Table 1.** Participant characteristics (N = 30).

Position	Number
**Clinical qualification**	
Paramedic (intern)	2
Paramedic	28
Intensive care paramedic	13
Special operations paramedic	2
Control/dispatch	2
Motorcycle	1
Rescue	1
**Managerial role**	
Line manager	3
Middle manager	2
Senior manager	3
**Location**	
Metropolitan	14
Regional/remote	16
**Length of service**	
1–5 years	7
6–10 years	8
11–20 years	8
21–30 years	5
31+ years	2
**Education**	
Vocational (diploma/grad) cert	23
Degree	13
Master’s	2
**Employment status**	
Full time	26
Part time	4

### Data analysis

The interview transcripts and reflective field notes were analysed thematically using NVivo (version 10) software. The coding strategy employed was both deductive and inductive, using existing research to build an *a priori* coding structure which was adjusted progressively to accommodate new information and themes identified by the researcher within the data. Once all data within a node had been analysed, the researcher created a detailed memo recording her interpretation of the most salient themes and insights which, together with the coded data, formed the basis of the research findings. Reflexivity was practised throughout the research process by critically reflecting on the potential impact of the researcher’s personal standpoint and characteristics on the collection, analysis and interpretation of interview data.

## Results

### Experiences of sex-based harassment

Overall, 25 of the 30 women interviewed reported having experienced one or more forms of sex-based harassment from male colleagues at some point in their career. Most frequently (n = 24), this was in the form of gender harassment, that is, comments and behaviours targeting the women’s gender which created a hostile and unwelcome environment (Berdahl, 2007). Sexual harassment was also experienced by some women (n = 4) in the form of unwelcome sexualised attention and overtures.

### Gender harassment

Interviews were replete with accounts of women being subjected to belittling comments and jokes about their suitability for and place within a profession which was, until 1979, performed exclusively by men. In many cases, the gender harassment came from senior and long-serving male paramedics:

There was a quote from one of the [managers] in our area; he uses this so many times. He said, ‘The day when females entered the service was the absolute worst day for the service’. (Paramedic 4)

Several younger participants spoke of having their skills and abilities questioned by colleagues who equated competency with age and time served ‘on the road’:

I struggled a lot with my colleagues as much as with the patients in terms of getting them to trust what I was saying and to trust my ability, despite the fact that I am young and female … It was a lot of jokes, ‘You don’t look like you’ve even finished high school’ or ‘Have you even got your license to drive the ambulance?’ (Paramedic 3)

However, being older and having substantial experience in the job did not necessarily insulate women from these kinds of remarks, as the accounts of these two highly experienced paramedics illustrated:

Even now, I get put down by the males … To the guys that we’re working with, it’s like ‘You will never be as good as we are. We’re the professionals’. Clinically, we’re doing the same job with the same outcomes, but they just constantly want to put you down. [It’s] very wearing. (Paramedic 13)

I still get sexist jokes like ‘What are you doing out of kitchen?’ (Paramedic 21)

Finally, several women had been subject to harassment that was both gendered and homophobic, pointing to a ‘double whammy’ of marginalisation that women paramedics who identified as LGBTQI+ could experience:

I’ve had a Duty Operations Manager mention in the last 6–12 months, ‘It was all good until the girls were let in the job, and now we just have lesbians trying to make out with each other everywhere we go and we can’t get anything done’ … he’s also not really pulled up or disciplined. (Paramedic 20)

### Sexual harassment

Several participants relayed their experiences of unwanted sexualised conduct from male colleagues. The most serious case involved a woman being harassed and stalked for a period of one year by a male co-worker, which she said began after she complained to her manager about the condition of the station’s toilets:

The cubicles would have [pornographic] photos inside them, they would have urine on the floor, urine on the seats and they were just disgusting. And I used to say, ‘We need a separate cubicle for the women’. When I started saying that this one guy kept following me into toilets – because there was no lock on the main door – every time I went to a cubicle, he’d go in to the one next door and ask me [sexually explicit question]. (Paramedic 13)

Upon reporting the predatory conduct to her manager, she was told that she could be transferred ‘to the country where there’s separate female toilets’, which enabled the behaviour to go ‘on and on and on’.

Other participants spoke about inappropriate sexual conduct from male training officers towards young women. One participant experienced the following after returning to work following a period of maternity leave:

I had my return-to-work training for two days and my training officer asked if I needed help expressing milk. He’s known for being a dirty perv[ert]. I told him I needed to take a lactation break and he said ‘OK, we’ll put you in this conference room because it can be locked. But let me know if you need a hand, hey?’ (Paramedic 1)

Another woman recalled her training instructor making ‘many comments that “All you had to do was lower your zipper a little bit on your shirt and you’d pass”’ (Paramedic 30). The suggestion that women could obtain a career advantage by exposing parts of their body played into a damaging stereotype of women ‘using’ their sexuality to advance their careers:

When the intensive care paramedic was advertised, I got through on my own merit. But [a male colleague] accused me of using my body to get what I want in the service … He said my reputation was that, oh, yeah, she’s got big boobs and that’s it. That’s it. (Paramedic 4)

### Responses to sex-based harassment

#### Barriers to reporting

Consistent with research showing that most sex-based harassment goes unreported, participants articulated a range of barriers to speaking out about harassment. The first related to the difficulty in gathering adequate evidence when the behaviour was perpetrated in a deliberately covert way:

He’s very, very careful about what he does. He doesn’t put anything in writing so it’s really hard, and what he says to one person is completely contradictory to what he’d say to another person and everything’s behind closed doors and it always comes down to ‘he said, she said’. (Paramedic 17)

There was also the risk that without sufficient evidence of the harassment, the woman could be perceived as overly sensitive or a ‘troublemaker’:

It was really difficult because it was that passive-aggressive, not-very-obvious stuff … very hard to report on, and when I used to try to talk to my manager about it, I felt like a whinger, because no one else could see what he was doing … He’d been in the job for almost 40 years [so] he knew what he was doing, and he knew how to do it and how to get away with it. (Paramedic 14)

One participant noted that there was a ‘huge joking culture’ which could create doubt in the victim’s mind about whether offensive behaviour should be defined as harassment:

There are always going to be those comments that get made and you have to work out, ‘is this a joke thing or is this overtly sexist?’ Like, do I pull them up on it or do I just kind of laugh one off? Would they say that to their wife or their daughter or is it because they’re at work that they say it? (Paramedic 20)

The second barrier to reporting harassment was a belief that the complaint would not be properly investigated and acted upon, particularly if the perpetrator was a manager. Many participants believed that there were informal ‘boys’ clubs’ in the organisation which insulated certain men from the consequences of their actions:

The higher levels of management protected him and ensured that he didn’t lose his job. I was told by someone that this particular man was a friend and had come up through the service with those in higher positions. And the old boys’ school – or whatever they call it – would make sure that they protected him. (Paramedic 15)

Finally, a fear that making a complaint could expose the woman to further harm was another reason women were hesitant to do so. Several participants described a general culture of silence surrounding the reporting of bullying and harassment based on an expectation of retaliation by the perpetrator:

There’s a lot of stuff that goes on that doesn’t get reported because, I guess, people – females in general – don’t want to draw attention to themselves and make it worse. Senior management don’t hear about it, so they think everything is okay … I think it’s just a fear of being bullied more – people don’t come forward. (Paramedic 25)

Confidentiality is not a great thing in the service. So if you speak up, they’re going to find out exactly who’s told who and who’s saying all of this and get repercussions from that. (Paramedic 30)

#### Informal coping strategies

The potential costs and consequences of reporting sex-based harassment meant that participants were more likely to adopt informal strategies to manage the behaviours and minimise the harm to their careers and well-being. Ignoring the behaviour was a common response and was seen as easier than calling the men out on their behaviour which could prompt further derision and mockery.

It’s easier not to engage. If you question it, you are framed very much as the angry female. (Paramedic 9)

Using humour was another way women attempted to respond to harassment, and could involve deflecting or joining in with sexist jokes to diffuse the situation:

The smart women will just make jokes back at them and, like, start highlighting, ‘Oh, yeah, but lucky I’m not a princess because otherwise this would be awkward’, or just start making jokes, like, ‘Oh, do you think you can go get this bag for me because I’m, you know, I’m a female and I can’t carry it?’ (Paramedic 19)

Several women said that they had directly appealed to the co-worker to change his behaviour but that this rarely resolved the issue and carried the risk of denial and further hostility:

I’d approached the guy a couple of times and I tried to have that straight talk face-to-face thing with him, ‘What’s your issue, do you have a problem with me?’ Every time ‘No, no issue with you, nothing’ and all the time I knew he was lying because he’d say stuff to other people and they’d be saying it to me and I’d ask him stuff and I’d be sitting there going ‘I know you’re lying to me’. (Paramedic 14)

Finally, some women who experienced serious or prolonged harassment sought relief by withdrawing from the work environment, either temporarily or permanently. One participant had taken an extended leave of absence following sustained bullying by male colleagues. A more lasting solution was offered by moving to a different ambulance station or to a non-frontline role within the organisation, a step this participant felt she had no choice but to take:

It just happened to work out there was a secondment that was coming up and so I applied for that and I was successful so that at least got me off the station … At the time I really didn’t know what I could do and I was such a mess that I actually considered taking my own life during that time … My only option was to get out of it, so that’s what I did. (Paramedic 25)

Others reported that they had considered leaving their jobs because of the harassment they experienced or had known women who had left the organisation for this reason.

## Discussion

A large majority of participants in this study had experienced sex-based harassment from male colleagues on one or more occasions in their careers, whether in the form of sexist comments and jokes, or unwanted sexualised conduct. Yet because of the covert and everyday nature of much of the harassment and a lack of confidence in the complaints-handling process, reporting sex-based harassment was considered by many as a last resort. This meant that the burden of managing these incidents was shouldered largely by the women themselves.

The findings of this study are significant in a number of respects. They offer much-needed insights into the workplace experiences of women in the paramedic profession, a topic which, hitherto, has received very little attention in the scholarly literature. While prior research has found women to be at high risk of sexual harassment and abuse (Bigham et al., 2014; Boyle et al., 2007; Staats et al., 2021), this study uses qualitative methods to delve behind these statistics, generating rich accounts of the women’s lived experiences of harassment, including the varied ways they attempted to cope with these troubling encounters. The findings build on the evidence regarding the hostile treatment of women in male-dominated emergency service professions (Gonsoulin & Palmer, 1998; Russ-Eft et al., 2008; Staats et al., 2021), pointing to the endurance of masculine cultures and ongoing resistance by some to women’s presence in paramedicine – despite their large share of the workforce. In doing so, the findings offer a possible explanation for why women remain under-represented in ambulance leadership roles and continue to report discrimination in their treatment at work (Mason et al., 2018; Staats et al., 2021).

Given the potentially dire consequences of sex-based harassment for victims and the significant flow-on costs for organisations, the study has important implications for policy and practice. Ambulance organisations have legal and ethical duties to ensure workplaces are safe for all employees, including taking reasonable steps to prevent and minimise the risk of sexual harassment (Safe Work Australia, 2021). Research suggests that this is best achieved by building an organisational climate which is not tolerant of sex-based harassment – that is, a climate in which victims are not discouraged from, or punished for, reporting harassment; complaints are taken seriously and investigated properly; and action is taken against those found to have perpetrated this conduct (Hulin et al., 1996).

At a time when women’s participation in the paramedic profession continues to grow, it is particularly important that employers examine how their workplace policies and practices may be inhibiting women’s inclusion and career advancement. The results of this study suggest that the mere presence of large numbers of women will not be sufficient to break down historically entrenched patterns of gender inequality.

### Limitations and future research

The study has limitations which open up possibilities for future research. Firstly, as the research sample was not statistically representative, the study’s findings cannot be generalised to broader populations of women paramedics. Notwithstanding this, the rich descriptions gathered of the women’s experiences mean that the findings are likely to have relevance and some degree of ‘transferability’ (Lincoln & Guba, 1985) to other organisational cases, particularly in Australia. Future survey-based or mixed-methods research using representative samples could establish the extent to which the experiences of the women in this study are shared more widely.

Secondly, while this study was focused on sex-based harassment perpetrated by staff members, we know that paramedics also experience very high rates of workplace violence from patients and other members of the public (Boyle et al., 2007; Maguire, 2018). There is a need for further research to advance our understanding of women’s experiences of sex-based harassment and gendered abuse from those outside the organisation.

Finally, the larger study from which this study was drawn examined only the workplace experiences of women paramedics and hence the perspectives of male paramedics are missing from the analysis. Future research could investigate men’s experiences of and views on sex-based harassment in paramedicine.

## Conclusion

Sex-based harassment is a significant but under-researched problem in the paramedic profession. The majority of participants in this qualitative study had been the targets of gender and/or sexual harassment; however, many were reluctant to report the behaviour, believing no action would be taken against the perpetrator – or worse, that retaliation and repercussions may follow a complaint. The study advances our knowledge about the kinds of sex-based harassment experienced by women paramedics and the responses they utilised in an effort to remedy the conduct and minimise its impact on their well-being and careers. The findings point to several fruitful avenues for future inquiry and highlight the need for targeted action by employers to address and prevent these harmful workplace behaviours.

## Acknowledgements

The author would like to thank all study participants for their time and generosity in sharing their experiences and insights in interviews. She would also like to express gratitude to her doctoral supervisor, Rae Cooper, for her expert guidance and support throughout the PhD programme. Thanks also to Stephen Clibborn for his helpful feedback on an earlier draft of this manuscript.

## Author contributions

SHO acts as the guarantor for this article.

## Conflict of interest

None declared.

## Ethics

Ethical approval was obtained from the University of Sydney’s Human Research Ethics Committee (Protocol number 2015/486) and the Sydney Local Health District Ethics Review Committee (RPAH Zone) (Protocol Number X15-0394).

## Funding

This research was supported by an Australian Government Research Training Program (RTP) Scholarship which assists with the living costs of research doctorate students.
